# Supplementary material for: A Psychosocial Intervention’s Impact on Quality of Life in AYAs with Cancer: A Post Hoc Analysis from the Promoting Resilience in Stress Management (PRISM) Randomized Controlled Trial
Source: Children (Basel). 2019 Nov 2;6(11):124. doi: 10.3390/children6110124 (PMC6915541; doi:10.3390/children6110124)
Supplement: Supplementary file 1 [file children-06-00124-s001.pdf]

Supplementary Table S1. Generic and Cancer-Specific Quality of Life at Baseline and 6 months.

PedsQL SF-15	At Baseline				At 6 Months			
	Usual Care		PRISM		Usual Care		PRISM	
	n	mean (SD)	n	mean (SD)	n	mean (SD)	n	mean (SD)
Total score	44	59 (21)	48	62 (16)	38	60 (19)	36	67 (15)
Age 12-17	32	58 (22)	35	60 (16)	26	60 (19)	27	66 (15)
Age 18-25	12	62 (21)	13	67 (14)	12	58 (20)	9	69 (16)
Newly diagnosed	30	62 (22)	38	60 (15)	27	60 (22)	30	65 (14)
Advanced cancer	14	53 (19)	10	67 (20)	11	59 (13)	6	73 (19)
Physical health summary	44	46 (29)	48	48 (29)	38	48 (32)	36	57 (29)
Age 12-17	32	45 (30)	35	47 (29)	26	52 (31)	27	53 (30)
Age 18-25	12	48 (27)	13	50 (30)	12	41 (33)	9	68 (27)
Newly diagnosed	30	51 (30)	38	45 (29)	27	51 (34)	30	57 (28)
Advanced cancer	14	36 (24)	10	60 (27)	11	43 (26)	6	57 (38)
Emotional functioning	44	60 (24)	48	70 (21)	38	63 (24)	36	73 (18)
Age 12-17	32	60 (24)	35	67 (21)	26	67 (24)	27	74 (18)
Age 18-25	12	58 (26)	13	78 (19)	12	55 (23)	9	72 (19)
Newly diagnosed	30	64 (23)	38	68 (21)	27	66 (25)	30	71 (18)
Advanced cancer	14	51 (25)	10	79 (19)	11	57 (19)	6	82 (14)
Social functioning	43	88 (16)	48	86 (16)	38	78 (20)	36	86 (17)
Age 12-17	31	87 (17)	35	85 (17)	26	76 (18)	27	89 (13)
Age 18-25	12	89 (11)	13	89 (11)	12	81 (24)	9	78 (24)
Newly diagnosed	29	87 (17)	38	86 (14)	27	75 (21)	30	85 (17)
Advanced cancer	14	88 (15)	10	86 (22)	11	84 (15)	6	94 (14)
School functioning	38	64 (27)	42	57 (27)	36	60 (27)	32	60 (26)
Age 12-17	27	60 (29)	33	52 (25)	24	56 (25)	25	56 (24)
Age 18-25	11	73 (22)	9	78 (25)	12	69 (29)	7	75 (29)
Newly diagnosed	26	66 (29)	34	58 (27)	25	59 (29)	26	59 (25)
Advanced cancer	12	60 (24)	8	53 (26)	11	63 (23)	6	68 (31)

PedsQL Cancer Module								
	n	mean (SD)	n	mean (SD)	n	mean (SD)	n	mean (SD)
Total score	44	65 (17)	48	66 (16)	38	64 (20)	36	72 (11)
Age 12-17	32	66 (17)	35	63 (16)	26	65 (18)	27	71 (12)
Age 18-25	12	64 (16)	13	73 (11)	12	60 (25)	9	76 (7)
Newly diagnosed	30	67 (19)	38	64 (15)	27	65 (22)	30	71 (10)
Advanced cancer	14	62 (11)	10	70 (17)	11	60 (17)	6	79 (17)

Pain and Hurt	43	58 (26)	48	52 (27)	38	60 (30)	36	59 (27)
Age 12-17	31	63 (24)	35	53 (27)	26	67 (26)	27	59 (27)
Age 18-25	12	46 (25)	13	48 (27)	12	45 (34)	9	60 (30)
Newly diagnosed	29	57 (26)	38	51 (26)	27	68 (28)	30	60 (24)
Advanced cancer	14	61 (25)	10	55 (31)	11	42 (29)	6	58 (40)
Nausea	44	55 (25)	48	63 (22)	38	58 (27)	36	76 (17)
Age 12-17	32	58 (23)	35	62 (23)	26	60 (28)	27	75 (16)
Age 18-25	12	49 (29)	13	66 (20)	12	55 (25)	9	80 (21)
Newly diagnosed	30	55 (26)	38	64 (22)	27	58 (27)	30	74 (17)
Advanced cancer	14	56 (23)	10	60 (21)	11	58 (27)	6	88 (14)
Procedural anxiety	44	76 (23)	48	65 (33)	38	77 (28)	36	73 (26)
Age 12-17	32	78 (20)	35	58 (33)	26	81 (22)	27	70 (23)
Age 18-25	12	69 (31)	13	83 (26)	12	69 (37)	9	81 (34)
Newly diagnosed	30	77 (25)	38	61 (33)	27	75 (32)	30	71 (27)
Advanced cancer	14	74 (19)	10	79 (30)	11	83 (17)	6	83 (23)
Treatment anxiety	44	81 (18)	48	80 (23)	38	73 (27)	36	86 (16)
Age 12-17	32	82 (18)	35	75 (25)	26	77 (22)	27	85 (16)
Age 18-25	12	78 (18)	13	95 (9)	12	65 (34)	9	88 (15)
Newly diagnosed	30	84 (18)	38	79 (23)	27	77 (27)	30	84 (16)
Advanced cancer	14	75 (16)	10	85 (24)	11	65 (25)	6	94 (14)
Worry	44	52 (26)	48	61 (23)	38	50 (29)	36	66 (22)
Age 12-17	32	56 (26)	35	59 (22)	26	52 (28)	27	65 (22)
Age 18-25	12	42 (25)	13	66 (26)	12	46 (33)	9	68 (21)
Newly diagnosed	30	59 (26)	38	61 (25)	27	54 (29)	30	63 (21)
Advanced cancer	14	37 (20)	10	62 (15)	11	41 (27)	6	76 (21)
Cognitive problems	43	65 (22)	48	63 (20)	38	64 (21)	36	69 (22)
Age 12-17	31	64 (24)	35	60 (19)	26	64 (19)	27	65 (22)
Age 18-25	12	68 (16)	13	70 (20)	12	64 (25)	9	79 (16)
Newly diagnosed	29	67 (23)	38	59 (19)	27	66 (21)	30	67 (21)
Advanced cancer	14	61 (19)	10	76 (17)	11	60 (20)	6	75 (26)
Perceived physical appearance	43	68 (29)	48	66 (24)	38	60 (30)	36	70 (23)
Age 12-17	31	64 (30)	35	63 (25)	26	56 (30)	27	70 (25)
Age 18-25	12	79 (22)	13	75 (19)	12	67 (29)	9	69 (20)
Newly diagnosed	29	71 (30)	38	67 (22)	27	58 (32)	30	71 (21)
Advanced cancer	14	63 (27)	10	65 (33)	11	63 (23)	6	68 (37)
Communication	43	72 (23)	48	74 (17)	38	68 (27)	36	80 (22)
Age 12-17	31	70 (24)	35	70 (17)	26	65 (27)	27	79 (21)
Age 18-25	12	78 (19)	13	83 (15)	12	72 (30)	9	86 (27)
Newly diagnosed	29	74 (22)	38	73 (18)	27	68 (31)	30	79 (23)
Advanced cancer	14	67 (24)	10	75 (15)	11	66 (19)	6	85 (22)

Supplementary Table S2. Internal Consistency of the Sample.

A. Cronbach's alpha for Generic Quality of Life Subscales

	Baseline	6-month follow up
**Physical**	0.91 (lower 95% CI: 0.88)	0.92 (lower 95% CI: 0.9)
**Emotional**	0.85 (lower 95% CI: 0.81)	0.84 (lower 95% CI: 0.78)
**Social**	0.71 (lower 95% CI: 0.61)	0.67 (lower 95% CI: 0.54)
**School**	0.86 (lower 95% CI: 0.8)	0.86 (lower 95% CI: 0.8)
**Total**	0.89 (lower 95% CI: 0.85)	0.86 (lower 95% CI: 0.82)

B. Cronbach's alpha for Generic Quality of Life Subscales

	Baseline	6-month follow up
**Pain**	0.85 (lower 95% CI: 0.79)	0.86 (lower 95% CI: 0.80)
**Nausea**	0.82 (lower 95% CI: 0.77)	0.87 (lower 95% CI: 0.83)
**Procedural anxiety**	0.89 (lower 95% CI: 0.86)	0.92 (lower 95% CI: 0.88)
**Treatment anxiety**	0.85 (lower 95% CI: 0.8)	0.89 (lower 95% CI: 0.85)
**Worry**	0.78 (lower 95% CI: 0.71)	0.81 (lower 95% CI: 0.73)
**Cognitive**	0.82 (lower 95% CI: 0.76)	0.84 (lower 95% CI: 0.79)
**Physical Appearance**	0.77 (lower 95% CI: 0.69)	0.75 (lower 95% CI: 0.65)
**Communication**	0.76 (lower 95% CI: 0.67)	0.93 (lower 95% CI: 0.9)
**Total**	0.91 (lower 95% CI: 0.89)	0.92 (lower 95% CI: 0.9)
